# The first record of leucism in the *Rhabdophis tigrinus* (Boie, 1826) (Squamata, Colubridae) in South Korea

**DOI:** 10.1002/ece3.11029

**Published:** 2024-02-22

**Authors:** Seung‐Min Park, Seung‐Ju Cheon, Hye‐Rin Park, Na‐Yeong Kim, Md Mizanur Rahman, Ha‐Cheol Sung

**Affiliations:** ^1^ Department of Biological Sciences Biotechnology Chonnam National University Gwangju South Korea; ^2^ Research Center for Endangered Species National Institute of Ecology Yeongyang South Korea; ^3^ Department of Biological Sciences Chonnam National University Gwangju South Korea

**Keywords:** abnormal color variation, ecdysis, normal eyes, tiger keelback, yellowish snake

## Abstract

Leucism, in which pigmentation is lost over part or the entire body of an animal, has a range of possible genetic causes. Here, we report leucism in an individual tiger keelback (*Rhabdophis tigrinus*) found on Jeung Island, Shinan‐gun, Jeollanam‐do, South Korea, during a survey of the distribution of reptiles in the area. The individual was observed sunbathing in the bushes next to a pond. This individual exhibited ecdysis, thus it considered that have normal feeding activity. Our report represents the first observation of leucism in *R*. *tigrinus*, and thus, further analysis is needed of this phenotype to more clearly understand its impact on the species and its natural history.

## Graphical abstract

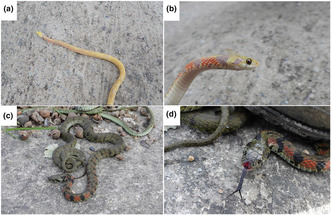

## 1 INTRODUCTION

Abnormal color variation on parts or over the entire body of animals has a variety of genetic causes (Ashaharraza & Lalremsangha, 2020). An example of this abnormal coloring is leucism, which typically presents as a partial loss of pigmentation (Lobo & Sreepada, 2016). It appears similar to albinism, but albino individuals tend to have red eyes due to a complete lack of pigmentation (Deshmukh et al., 2020), while individuals with leucism have normal‐colored eyes (Acevedo & Aguayo, 2008). Animals with abnormal color variation, such as those with leucism, can be disadvantaged (potential problems with thermoregulation and visual acuity) in nature in various ways (Bruni, 2017; Krecsák, 2008) thus, it may encounter an increased risk of predation (Di Marzio & Rozentāls, 2021). For these reasons, due to low survival rate, individuals with leucism are difficult to observe in nature (Lobo & Sreepada, 2016).

The tiger keelback (*Rhabdophis tigrinus*; Boie, 1826) is a species of snake found in Russia, Japan (Lee et al., 2011), and throughout South Korea, including Jeju Island (Jang et al., 2016). This snake is a venomous snake and has two venom glands, which are used for digestion and defense (Hutchinson et al., 2007; Lee et al., 2011). The morphological characteristic of this snake is green with red and black spots on its neck and keeled on all over the scales (Lee et al., 2011).

Commonly, *R*. *tigrinus* is easily found in nearby grasslands, wetlands, and ponds (Lee et al., 2011). Nevertheless, herein, we report an individual of *R*. *tigrinus* with an abnormal color variation. Additionally, we build on the research review, collect data on leucism and albinism found in the species and genus.

## 2 MATERIALS AND METHODS

**FIGURE 1:**
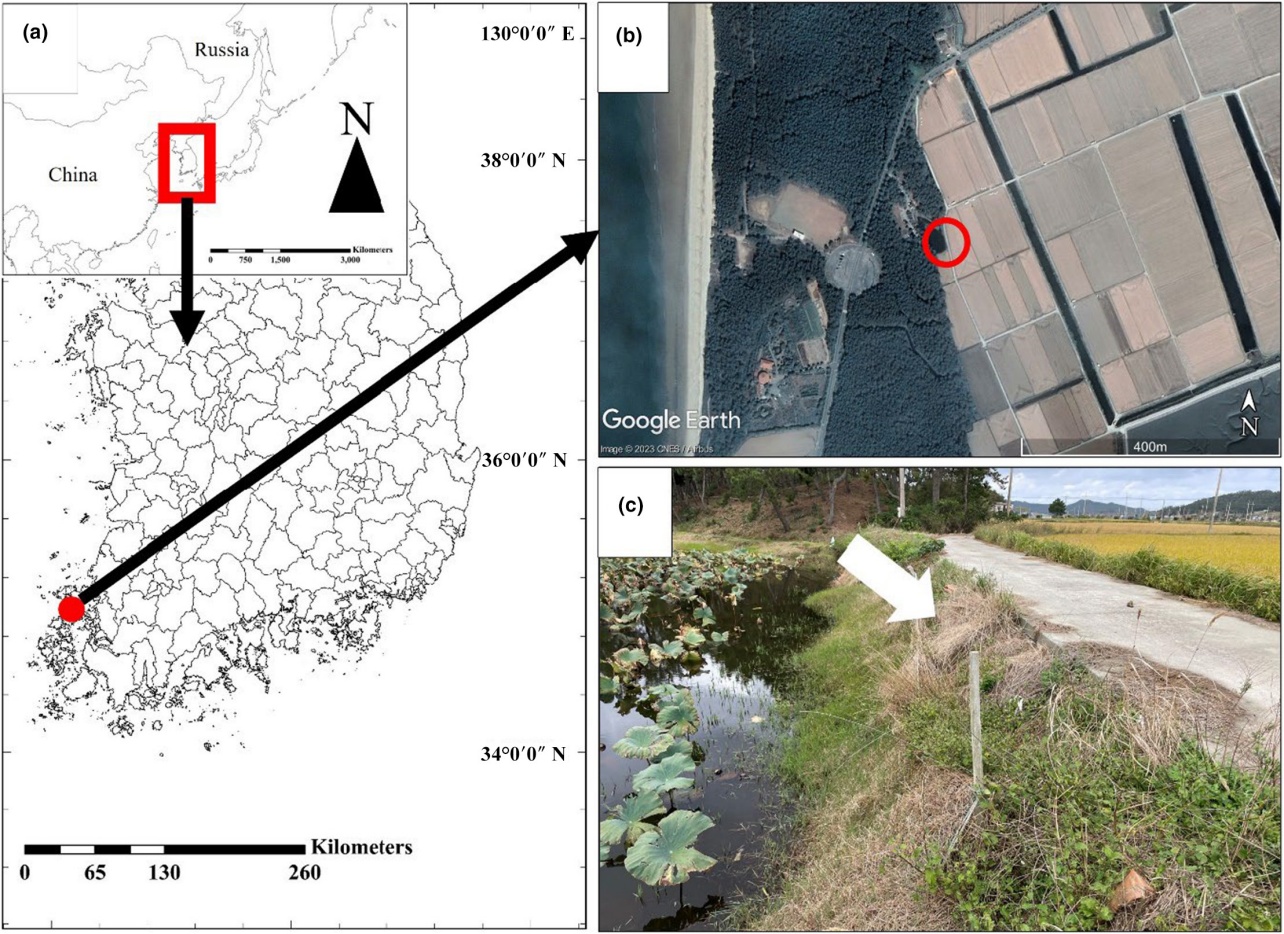
Location of the observed leucistic *Rhabdophis tigrinus* individual. (a) Satellite view. The red circle indicates where the individual was found, surrounded by agricultural land, a beach, a two‐lane road, a river, and a small pond. (b) Foreground photograph of a. (c) The white arrow indicates where we found the snake. The snake was sunbathing on the grass.

On October 10, 2022, we conducted a survey on the distribution of reptiles in Jeung Island (Shinan‐gun, Jeollanam‐do, South Korea; Figure 1). The region contains agricultural land, beaches, two‐lane roads, rivers, and a small pond. We observed reptile species while walking slowly across the road. We observed five species of reptile: *Scincella vandenburghi*, *Elaphe dione*, *Oocatochus rufodorsatus*, *R*. *tigrinus*, and *Hierophis spinalis*. We observed four *R*. *tigrinus* individuals (including one that was dead), but one had an abnormal color. The unusually colored individual was found near a small pond (34.971606° N, 126.140892° E, elevation 6 m, Figure 1). The individual was sunbathing in the bushes next to the pond. We caught the snake carefully, measured its body and tail length and after took photographs for identification, released it at the same location.

On online searches, we use two academic search platforms(Google scholar, www.scholar.google.co.kr; Scopus, www.scopus.com) and use the keywords: *Rhabdophis tigrinus*, leucism, albinism, tiger keelback, abnormal color variation, snake, Family Colubridae, Genus *Rhabdophis*. We searched by combining two or more words (leucism *Rhabdophis tigrinus*, abnormal color variation snake, etc.). Beside English, consider the distribution of this species, online searches were conducted in Japanese, Chinese, Russian, and Korean. Also, online search results were classified only from Southeast Asia and Middle East Asia.

## 3 RESULTS

**FIGURE 2:**
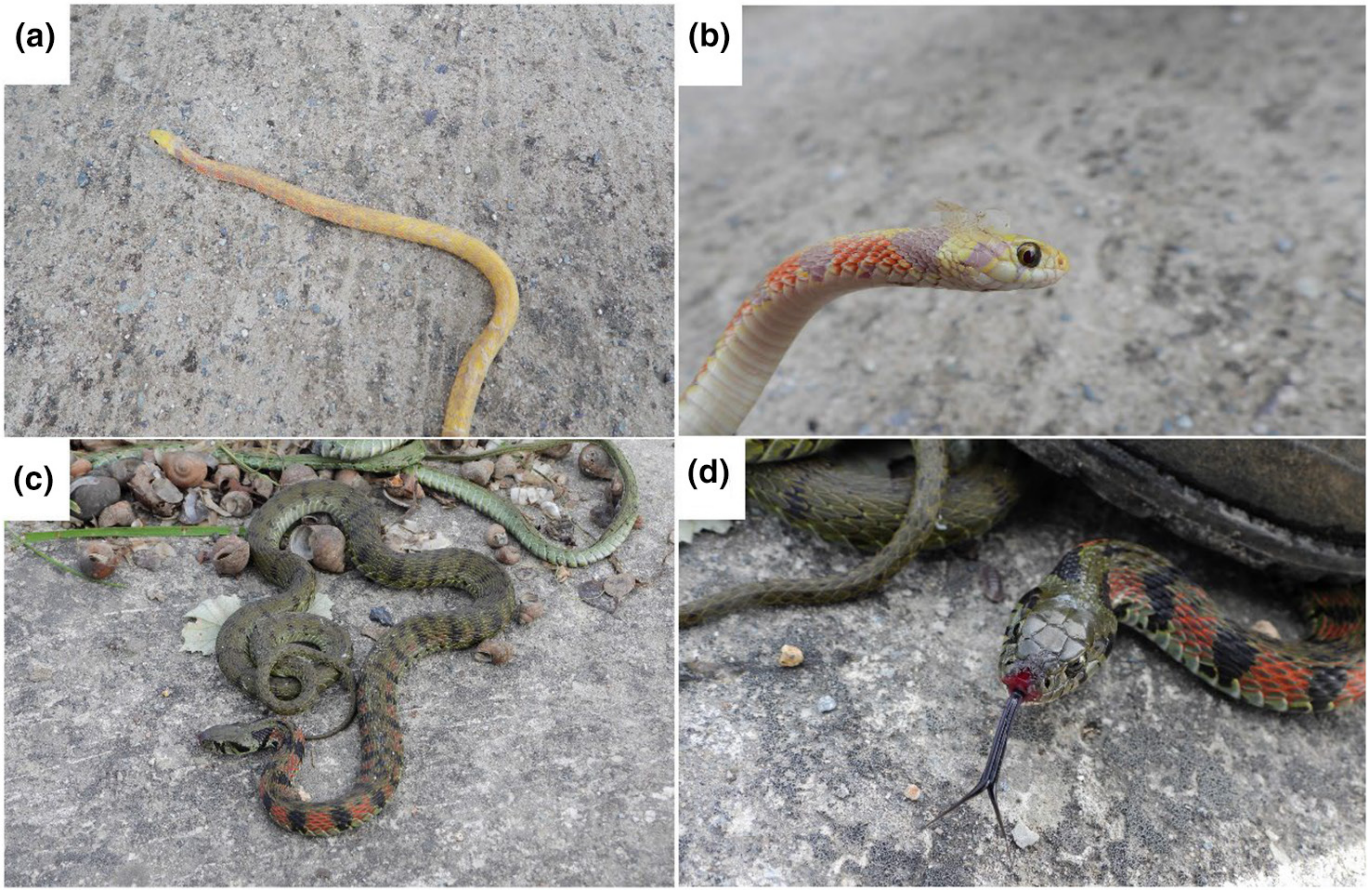
Leucistic and normal *Rhabdophis tigrinus* individuals observed in the survey. (a) Leucistic *R*. *tigrinus* individual showing an overall yellowish body color. (b) Close‐up of the head, showing that the originally black spots appear pink and that the eye color is normal. (c) Normal *R*. *tigrinus* individual exhibiting a greenish overall body color. (d) Close‐up of the head, showing red and black spots on the neck and a black tongue (The red coloring of the lips appears to be the result of injury and scarring). Photographs by Seung‐Min Park.

The observed individual was yellowish overall with no green or black color (Figure 2a), its pupils were the same color as those of normal individuals (Figure 2b), and it had a pink tongue. Its body length was 39.5 cm, while its tail length was 9.5 cm, for a total of 49 cm. Especially, we confirmed that the snake was during the process of ecdysis (Figure 2b). Based on its body pattern and morphological characteristics, this abnormal individual was identified as *R*. *tigrinus*, while the characteristics of its abnormal color variation (i.e., body, eyes, and tongue) suggested that it was leucistic (Lobo & Sreepada, 2016; Urra et al., 2021). Another normal *R*. *tigrinus* individual observed in the same location was greenish overall, with black spots across its entire body and a black tongue (Figure 2c,d).

We found total 11 species of snake of leucism and albinism and location were mostly India (Table 1). However, we found no reports of leucism and albinism with *R*. *tigrinus*. On the other hand, the same genus, we found that albinism of *R*. *rhodomelas* and leucism of *R*. *plumbicolor* in Singapore and India.

**TABLE 1:** List of data recording albinism and leucistic snakes in Southeast Asia and Middle East Asia.

No.	Species	Type	Location	Year	References
1	*Rhabdophis rhodomelas*	Albinism	Singapore	2023	Foenander and Charlton (2023)
2	*Rhabdophis plumbicolor*	Leucistic	India	2014	Deshmukh et al. (2020)
3	*Fowlea piscator*	Leucistic; Albinism	India	2019; 2017	Ashaharraza and Lalremsangha (2020) and Deshmukh et al. (2020)
4	*Python molurus molurus*	Leucistic	India	2016	Lobo and Sreepada (2016)
5	*Bungarus caeruleus*	Leucistic; Albinism	India	2007, 2012, 2015, 2018; 2017	Mukherjee and Mohan (2021), Chaudhuri et al. (2018), and Deshmukh et al. (2020)
6	*Eryx conicus*	Albinism	India	2017, 2019; 2018	Mukherjee and Mohan (2021) and Deshmukh et al. (2020)
7	*Naja naja*	Albinism	India	2016, 2019	Mukherjee and Mohan (2021)
8	*Lycodon aulicus*	Albinism; Leucistic	India	2016; 2018	Mukherjee and Mohan (2021) and Deshmukh et al. (2020)
9	*Coelognathus helena*	Albinism	India	2011	Mukherjee and Mohan (2021)
10	*Ptyas mucosa*	Albinism	India	2019	Mukherjee and Mohan (2021)
11	*Oligodon arnensis*	Albinism	India	2017	Deshmukh et al. (2020)

## 4 DISCUSSION

*Rhabdophis tigrinus* is very common in South Korea and Japan (Lee et al., 2011), but reports of leucism have been rare. We searched online to find reported cases of albino and leucistic *R*. *tigrinus* but were unable to find any. We only found the same genus snakes in albinism and leucism (*R*. *rhodomelas* and *R*. *plumbicolor*). Especially, most of the albinism and leucism result were from India thus, this report has a significance as a very rare and important case as Chinese, Japanese, and South Korea. Therefore, this report is the first in all distribution of this species. In addition, abnormal color individuals are known to mainly occur in isolated environments (Krecsák, 2008; Tsuchihashi et al., 2011), so reporting on abnormal individuals to need to continue for population conservation.

Generally, the size of an adult *R*. *tigrinus* is 60–100 cm (Lee et al., 2011) thus, the observed individual was considered to not yet be fully mature. In reptiles, feeding and digestion are influenced by body temperature, and previous studies have suggested that individuals with a bright body color due to a lack of pigment may have difficulty in thermoregulating (Kornilios, 2014), with dark colors more efficient in terms of maintaining the body temperature (Gibson & Falls, 1979). For this reason, leucistic or albino individuals may experience harmful consequences for their survival and fitness due to the lack of thermoregulatory efficiency and camouflage (Krecsák, 2008). However, this individual seems to act normal.

Nevertheless, this individual appeared to be engaged in normal feeding activity because it was undergoing ecdysis (King & Turmo, 1997). As in this report, about the normal activity of individuals with leucism, Krecsák (2008) argued that this was possibly due to partially hidden the body as the trade‐off thermoregulation. Furthermore, Bruni (2017) suggested that they were mainly active around stagnant water with a temperature higher than the surrounding temperature at night. In fact, our observed individual was also found near a stagnant pond. However, the implications of these rare phenotypes for the metabolism, thermoregulation, and environmental fitness of affected individuals remain largely unknown (Urra et al., 2021). Therefore, further studies are needed to understand the impact of these abnormal phenotypes on the species and their natural history.

## AUTHOR CONTRIBUTIONS

**Seung‐Min Park:** Investigation (lead); methodology (lead); project administration (equal); writing – original draft (lead). **Seung‐Ju Cheon:** Investigation (equal). **Hye‐Rin Park:** Investigation (equal). **Na‐Yeong Kim:** Funding acquisition (lead); project administration (equal). **Md Mizanur Rahman:** Conceptualization (lead); writing – review and editing (equal). **Ha‐Cheol Sung:** Supervision (lead).

## FUNDING INFORMATION

This study was carried out with the support of NIE‐Entrusted Research‐2022‐56, 2022 National Distribution Survey of Endangered Wildlife (6th Phase 1st Year).

## CONFLICT OF INTEREST STATEMENT

All authors have no conflicts of interest in this study.

## Data Availability

We confirm that the Data Availability Statement is included in the manuscript and that access to all necessary data files is provided to editors and reviewers.
